# ArcR contributes to tolerance to fluoroquinolone antibiotics by regulating *katA* in *Staphylococcus aureus*

**DOI:** 10.3389/fmicb.2023.1106340

**Published:** 2023-02-24

**Authors:** Tongtong Fu, Zheng Fan, Yujie Li, Zhoufei Li, Bing Du, Shiyu Liu, Xiaohu Cui, Rui Zhang, Hanqing Zhao, Yanling Feng, Guanhua Xue, Jinghua Cui, Chao Yan, Lin Gan, Junxia Feng, Ziying Xu, Zihui Yu, Ziyan Tian, Zanbo Ding, Jinfeng Chen, Yujie Chen, Jing Yuan

**Affiliations:** ^1^Department of Bacteriology, Capital Institute of Pediatrics, Beijing, China; ^2^Department of Life Science and Medicine, University of Science and Technology of China, Hefei, China; ^3^Military Supplies and Energy Quality Supervision Station of NV, PLA, Beijing, China

**Keywords:** *Staphylococcus aureus*, ArcR, fluoroquinolone antibiotics, oxidative stresses, catalase, KatA

## Abstract

*Staphylococcus aureus* is an opportunistic pathogen that shows a unique ability to quickly respond to a variety of antibiotics. The Crp/Fnr family transcriptional regulator ArcR controls expression of arginine deiminase pathway genes *arcABDC*, which enable the utilization of arginine as an energy source for cell growth under anaerobic conditions. However, ArcR shares low overall similarity with other Crp/Fnr family proteins, suggesting that they differ in the response to environmental stress. In this study, MIC and survival assays were performed to determine the role of ArcR in antibiotic resistance and tolerance. The results showed that deletion of *arcR* reduced tolerance of *S.aureus* to fluoroquinolone antibiotics, mainly through a defect in the response to oxidative stress. In *ΔarcR* mutant, the expression of the major catalase gene *katA* was downregulated, and *katA* overexpression restored bacterial resistance to oxidative stress and antibiotics. We showed that ArcR directly regulated *katA* transcription by binding to the promoter region of *katA*. Therefore, our results revealed the contribution of ArcR in bacterial tolerance to oxidative stress and subsequently to fluoroquinolones antibiotics. This study added our understanding on the role of Crp/Fnr family in bacterial susceptibility to antibiotics.

## Introduction

*Staphylococcus aureus* is one of the most common pathogens worldwide and causes a variety of diseases from mild skin infection to fatal endocarditis and sepsis (Levy and Marshall, 2004; Cheung et al., 2021). Antibacterial therapy is one of the only curative pharmacological treatments. Antibiotics exert a bacteriostatic or bactericidal effect by interacting with their targets, *via* three main mechanisms: inhibition of DNA replication or RNA transcription; disruption of protein synthesis; and prevention of cell wall renewal (Walsh, 2000). Several studies showed that aminoglycoside, β-lactam, and quinolone antibiotics, regardless of drug-target interactions, could stimulate the formation of highly deleterious hydroxyl radicals that assist the bactericidal process (Imlay and Fridovich, 1991; Dwyer et al., 2007; Kohanski et al., 2007). It was well accepted that majority of superoxide generation in *E. coli* occurs through oxidation of the respiratory electron transport chain driven by oxygen and the conversion of NADH to NAD^+^. NADH is generated from NAD^+^ during the TCA cycle. Aminoglycoside, β-lactam, and quinolone antibiotics could result in catabolic NADH depletion, which facilitated the strengthening of the TCA cycle and the generation of superoxide such as hydrogen peroxide (H_2_O_2_). Then, superoxide could stimulate the Fenton reaction that produced hydroxyl radicals (Imlay and Fridovich, 1991; Dwyer et al., 2007). All three of these types of bactericidal antibiotics stimulate the production of highly harmful reactive oxygen species (ROS) in both Gram-negative and Gram-positive bacteria, which ultimately help to kill bacteria (Kohanski et al., 2007; Singh et al., 2021).

In natural or host immune system environment, bacteria respond to various reactive species that could modify the bacterial proteome by post-translational modifications and result in bacterial defense through dedicated signal transduction pathways. The reactive species include ROS that are generated in bacteria as the unavoidable consequence of the aerobic life, by incomplete reduction of molecular oxygen during respiration (Imlay, 2003, 2008). The ROS are composed by superoxide anion O_2_•^−^, H_2_O_2_, and the highly reactive OH•. Catalases and peroxidases are the major peroxide scavengers that convert H_2_O_2_ to H_2_O and O_2_ through different mechanisms. Catalases have an extremely high peroxide turnover rate and make function at high H_2_O_2_ concentrations. In *S. aureus*, the major catalase KatA is present that is peroxide-inducible and confers H_2_O_2_ resistance. Mutations in the gene encoding catalase increase antibiotic susceptibility in *Burkholderia*. In *Pseudomonas aeruginosa*, mutation of *katA* results in increased susceptibility to ciprofloxacin, aminoglycosides, and β-lactam antibiotics (Yu and Deutscher, 1995).

Members of the Crp/Fnr family are DNA-binding proteins that function as transcriptional regulators which primarily activate the expression of genes, although some also repress the expression (Körner et al., 2003). ArcR belongs to the Crp/Fnr family regulators, whose gene *arcR* and arginine deiminase pathway genes *arcABDC* form an operon in *S. aureus*. There was 19% identity and 45% similarity between the ArcR open reading frame of *S. aureus* and *Bacillus licheniformis*, but less similarity compared with Crp of *Escherichia coli*. Under anaerobic conditions, ArcR binds to the upstream regulatory regions of *arcABDC* to positively regulate their transcription, and this process can be influenced by glucose and arginine (Makhlin et al., 2007). Apart from this regulatory process, the role of ArcR in the response of *S. aureus* to environmental stress, such as antibiotics, remains unknown.

In this study, we found that ArcR played an important role in the resistance of *S. aureus* to fluoroquinolones antibiotics. When *arcR* was deleted, *S. aureus* had a reduced response to oxidative stress. We further demonstrated that ArcR positively regulated the transcription level of *katA* by directly binding to the promoter region of *katA*, thereby affecting the tolerance of bacteria to oxidative stress and fluoroquinolone antibiotics.

## Materials and methods

### Bacterial strains, plasmids, and growth conditions

The bacterial strains and plasmids used in this study are listed in Table 1. The primers used are listed in Table 2. When necessary, the corresponding antibiotics (100 μg/ml ampicillin and 50 μg/ml kanamycin) were added to the medium. *S. aureus* was usually cultured in Tryptone Soy Broth medium at 37°C and 220 rpm, to which 15 μg/ml chloramphenicol and 10 μg/ml erythromycin were added when necessary.

**Table 1:** Bacterial strains and plasmids used in this study.

Strain or plasmid	Relevant characteristic	Source or reference
*S. aureus*		
RN4220	8,325–4, r^−^, restriction-deficient mutagenized RN450	(Kreiswirth et al., 1983)
NCTC8325 WT	NCTC8325 wild-type strain	NASA^a^
NCTC8325 Δ*arcR*	NCTC8325 *arcR* deletion mutant	This study
*E. coli*		
DH5α	*E. coli* host for cloning	Vazyme
BL21(DE3)	Express strain; F^−^ *ompT hsdS*_B_ (r_B_^−^ m_B_^−^) *gal dcm* (DE3)	Vazyme
Plasmid		
pBTs	*E. coli/S. aureus* temp-sensitive plasmid, Apr^b^, Cmr, for the construction of allelic-exchange mutants	(Bae and Schneewind, 2006)
pBTs-*arcR*	pBTs derivative, for *arcR* deletion	This study
pCN51	*E. coli/S. aureus* expression vector, Apr^b^, Emr	(Charpentier et al., 2004)
pCN51-*arcR*	Inducible expression *arcR* in *S. aureus*	This study
pCN51-*katA*	Inducible expression *katA* in *S. aureus*	This study
pET28a	Expression vector with His tag in *E. coli*, Kmr	Addgene
pET28a-*arcR*	His6-ArcR expression vector	This study

a NARSA, network on antimicrobial resistance in *S. aureus*.

^b^ Ap, ampicillin; Cm, chloramphenicol; Em, erythromycin; Km, kanamycin.

**Table 2:** Primers used in this study.

Name	Sequence (5′-3′)	Purpose
RarcR-UP-F-EcoRI	ATCGCAGTGCAGCGGAATTCTTGTGGTGCAATGTCACAGGGTATG	*arcR* deletion
RarcR-UP-R	TGTTTGCTTACTAACCAATCTGCTAATGCCTTTAGTTCATG	
RarcR-DN-F	CATGAACTAAAGGCATTAGCAGATTGGTTAGTAAGCAAACA	
RarcR-DN-R-HindIII	AAACTACCGCATTAAAGCTTAGCATCAGCAGCATTTACTACCG	
pCN51-*arcR*-F	GGTCAATGTCTGAACCTGCAG AGTTGTCTGCTGACACTTTGC	Expression of *arcR* in *S. aureus*
pCN51-*arcR*-R	TCCTCTAGAGTCGACCTGCAGTACGTTAGACCTCATGTTCAAC	
pCN51-*katA*-F	GGTCAATGTCTGAACCTGCAGATGTGTCTTGAGTTAAGACTACG	Expression of *katA* in *S. aureus*
pCN51-*katA*-R	TCCTCTAGAGTCGACCTGCAGTCTCTTGTTAGGAATCTTTACG	
pET28a-*arcR*-F	TTGAAGGAGTTTAACTTATGCATCATCATCATCATCACACAGAAAACTTTATTTTGG	Expression of
pET28a-*arcR*-R	TCCTCTAGAGTCGACCTGCAGTACGTTAGACCTCATGTTCAAC	*arcR* in *E. coli*
Q-16S rRNA-F	ACAAAGTGACAGGTGGTGCA	qRT-PCR
Q-16S rRNA-R	GTTTGTCACCGGCAGTCAAC	
Q-*perR*-F	ACTCATCCAACAGCTGATG	qRT-PCR
Q-*perR*-R	TCGAATCGACTTGATGAGTCTCC	
Q-*katA*-F	TAGACCAGTCCCAATTGCACCACC	qRT-PCR
Q-*katA*-R	TCGCTTTACAGTACTAAATAGTTATTG	
Q-*ahpC*-F	AGGTTCTTGGAGCGTAGTATGC	qRT-PCR
Q-*ahpC*-R	TGTGTACGAAGTGAGTATCAGTTG	
Q-*ahpF*-F	AAGAAACAGGTGTAACATTTGC	qRT-PCR
Q-*ahpF*-R	TGCTTGAACGACATCAGGAC	
Q-*dps*-F	CGGTAGGAGGAAACCCTGTA	qRT-PCR
Q-*dps*-R	TGATACATCATCGCCAGCAT	
Q-*hmp*-F	AAGGCTATATTGGCGCTGAA	qRT-PCR
Q-*hmp*-R	TGCAACGCTTAGTCTTGGAA	
Q-*norA*-F	TTCACCAAGCCATCAAAAAG	qRT-PCR
Q-*norA*-R	CTTGCCTTTCTCCAGCAATA	
Q-*norB*-F	TTGCAACGCTTTTAGGTTGG	qRT-PCR
Q-*norB*-R	TACACCTAATTCTGATCC	
Q-*srrA*-F	GTCATTTAGCAGAACATGGG	qRT-PCR
Q-*srrA*-R	ACAGGTCATACCTCCCACAC	
E-*arcR*-F	ATCTTCCATATTTAGTCTCC	EMSA
E-*arcR*-R	ACACCAGTTAACTTTTTGTCTTG	
E-*tetA*-F	AGCGCGTGTTGTTATGTCGC	EMSA
E-*tetA*-R	ACCTCCTTATTAGACAATGTG	

### Construction of knockout and complementary strains

To construct *arcR* knockout plasmid, the upstream and downstream homologous arm fragments were amplified from wild-type strain NCTC8325 and then ligated by overlap PCR. The obtained fragment was cloned into the pBTs plasmid digested with *Eco*R I and *Hin*d III. Ampicillin was used to screen the correct transformants. The pBTs derivative pBTs-*arcR* was electroporated into RN4220 for modification and transformed into NCTC8325 for allelic replacement as described (Hu et al., 2015). PCR and sequencing were used to verify the mutation. For the construction of complementary plasmids, *arcR* and *katA* genes were amplified from NCTC8325, and the obtained fragments were ligated into pCN51 plasmid digested with *Pst* I. The derivates pCN51-*arcR* and pCN51-*katA* were transformed into RN4220 and subsequently electroporated into Δ*arcR* mutant. Expression of *arcR* and *katA* was induced by 10 μM CdCl_2_ if necessary.

### Minimal inhibit concentration and survival assays

The MICs of *S. aureus* to a variety of antibiotics were determined in Mueller-Hinton Broth medium as described previously (Borrero et al., 2014). For the determination of time-dependent bactericidal curves, overnight culture was diluted into fresh TSB medium at a ratio of 1:100 and incubated at 37°C and 220 rpm until OD_600_ reached 1.0. The MICs of ciprofloxacin, ofloxacin, norfloxacin, levofloxacin, moxifloxacin, and garenoxacin were 0.32, 0.25, 0.48, 0.19, 0.125, and 0.03 μg/ml, respectively. Thiourea (150 mM) was added when the strains grew to OD_600_ 0.6. Samples were taken out at certain time points, diluted 10-fold, and colonies counted by dropping plate.

### H_2_O_2_ susceptibility assay

The overnight cultures were diluted with fresh TSB medium to OD_600_ 0.05 and then, H_2_O_2_ at a final concentration of 0.4 mM was added to the cultures as described previously (Oogai et al., 2016). They were incubated at 37°C with 150 rpm and 1 ml aliquots were removed at the indicated times. The supernatant was removed by centrifugation and pellet was washed twice with sterile 1 × PBS. The precipitates were diluted 10-fold and then dropped onto TSB plates. The plates were cultured overnight at 37°C, and colony counts were performed on the next day.

### Neutrophil extraction and ROS measurement

The whole blood of mice was collected with an anticoagulant tube, and neutrophils were extracted using a mouse peripheral blood neutrophil isolation kit (Solarbio, Beijing, China). The concentration of extracted cells was counted by cell counter. Measurement of ROS levels was determined as previously described with minor modifications (Wu et al., 2009). Neutrophil cells were diluted to 10^5^/ml using warm Hanks’ balanced salt solution (HBSS) containing 100 mM luminol and 1 U/ml horseradish peroxidase. The diluted cell suspension was added to a 96-well plate at 200 μl/well, and the reaction was performed at 37°C for 10 min. The bacterial strains were added at a multiplicity of infection (MOI) of 5. At the same time, 10^6^ cells were removed and infected with an MOI of 10. The reaction was carried out in a cell incubator at 37°C, and 20 μl reactant was removed every hour for dilution and dropping plate counting, to which 100 mM NAC was added if necessary.

### RNA extraction, reverse transcription, and quantitative real-time PCR

When the bacteria were cultured in TSB medium to OD_600_ 1.0, they were treated in PBS containing 0.4 mM H_2_O_2_ for 10 min and collected by centrifugation. Total RNA was extracted using RNAprep Pure Cell/Bacteria Kit (Tiangen Biotech, Beijing, China). Then, cDNA was synthesized with 1 μg RNA and PrimeScript™ IV 1st strand cDNA Synthesis Mix (Takara, Dalian, China). For the qRT-PCR, cDNA was mixed with specific primers (Table 2) and chamQ universal SYBR quantitative RT-PCR master mix (Vazyme, Nanjing, China). 16S rRNA gene was used as internal controls.

### Catalase activity assay

Bacteria were cultured to OD_600_ 1.0 using TSB medium, and 1 ml of culture was collected by centrifugation and washed twice with PBS. The cultures were incubated in PBS with or without 0.4 mM H_2_O_2_ for 30 min. The reaction samples were collected by centrifugation, washed twice with PBS, and broken by Ultrasonic Cell Disruptor (Xinzhi, Ningbo, China). Catalase activity was detected using a catalase assay kit (Beyotime, Shanghai, China).

### ArcR protein expression and purification

For the construction of ArcR expression plasmid, the *arcR* gene fragment was amplified from NCTC8325 genome and the PCR product was ligated with pET28a digested with *Eco*RI. Kanamycin was used to screen the correct transformants. PCR and sequencing were used for verification to obtain the correct expression of pET28A-his6-ArcR. The correctly constructed pET28a-His6-ArcR plasmid was transferred into *E. coli* BL21(DE3). The transformers were grown in LB medium containing 50 μg/ml kanamycin at 37°C until OD_600_ reached 0.6 and were induced with 0.5 mM IPTG at 37°C for 4 h. The cells were harvested and lysed by sonication with lysis buffer (25 mM HEPES, 5 mM β-mercaptoethanol, 500 mM NaCl, and 10% glycerol, pH 7.8). The His-tagged fusion proteins were purified in nickel nitrogen triacetate agarose solution (Qiagen, Beijing, China). The bounding protein was eluted by elution buffer (25 mM HEPES, 5 mM β-mercaptoethanol, 500 mM NaCl, 10% glycerol, and 500 mM imidazole, pH 7.8). Target protein was stored at −80°C until use. The concentration of purified proteins was determined by SDS-PAGE and bicinchoninic acid as the standard protein.

### Electrophoretic mobility shift assay

EMSA was performed as previously described with minor modifications (Hellman and Fried, 2007). We used NCTC8325 as the template to amplify *katA* promoter region DNA fragment. The 30 ng DNA fragment was incubated with 0, 100, 200, and 400 ng purified ArcR in binding buffer (25 mM HEPES, 1 mM dithiothreitol, 200 mM NaCl, and 10% glycerol, pH 7.8) at 37°C for 30 min. The 8% polyacrylamide gel was pre-electrophoresed in 1× Tris-borate-EDTA buffer (0.044 M Tris, 0.044 M boric acid, and 0.001 M EDTA, pH 8.0) for 1 h to remove impurities. After adding the sample, electrophoresis was performed for 1 h 40 min on ice. At the end of the electrophoresis, the glue was stained with 0.5 μg/ml ethidium bromide. Imaging was performed using a gel imager (Bio-Rad, Hercules, CA, USA).

## Results

### ArcR is involved in tolerance of *Staphylococcus aureus* to fluoroquinolone antibiotics

**Figure 1:**
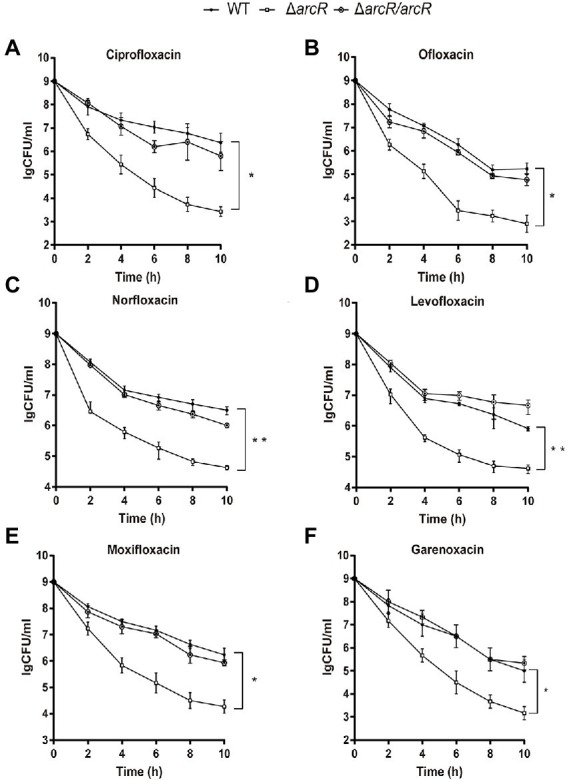
Roles of ArcR in bacterial resistance to fluoroquinolone antibiotics. Bacteria at OD_600_ 1.0 were treated with **(A)** 0.32 μg/ml ciprofloxacin, **(B)** 0.5 μg/ml ofloxacin, **(C)** 0.5 μg/ml norfloxacin, **(D)** 0.5 μg/ml levofloxacin, **(E)** 0.2 μg/ml moxifloxacin, or **(F)** 0.06 μg/ml garenoxacin in TSB at 37°C with agitation. Bacteria were collected at indicated times, and CFUs were determined by serial dilution and plating. Δ*arcR*/*arcR* represented the complementary strain, pCN51-*arcR* in Δ*arcR* mutant. Error bars represent standard deviations. The data represent results from three independent experiments. *p < 0.05, **p < 0.01, and ***p < 0.001, by Student’s *t*-test.

To understand whether ArcR affected antibiotic resistance of *S. aureus*, the MICs of Δ*arcR* mutants to ciprofloxacin, ofloxacin, linezolid, tetracycline, vancomycin, erythromycin, chloramphenicol, norfloxacin, levofloxacin, moxifloxacin, garenoxacin, and daptomycin were determined. Wild-type NCTC8325 and Δ*arcR* mutant displayed similar levels of resistance (MICs) to the above antibiotics (Table 3). However, following treatment with 0.32 μg/ml ciprofloxacin, 0.5 μg/ml ofloxacin, 0.5 μg/ml norfloxacin, 0.5 μg/ml levofloxacin, 0.2 μg/ml moxifloxacin, or 0.06 μg/ml garenoxacin, the survival rate of Δ*arcR* mutant was approximately 342-, 158-, 74-, 20-, 93-, or 87-fold lower than that of wild-type NCTC8325 strain after 10 h sterilization (Figures 1A–F).

**Table 3:** MICs of *S. aureus* to antibiotics.

Strain	MIC (μg/ml)					
	CIP	OFX	TCY	VAN	ERY	CHL
WT	0.63	1.25	1.25	0.63	0.25	0.63
Δ*arcR*	0.63	1.25	1.25	0.63	0.25	0.63
Δ*arcR/arcR*	0.63	1.25	1.25	0.63	0.25	0.63
	NOR	lEV	MFX	GRN	DAP	LZN
WT	0.48	0.19	0.125	0.03	1.25	0.13
Δ*arcR*	0.48	0.19	0.125	0.03	1.25	0.13
Δ*arcR/arcR*	0.48	0.19	0.125	0.03	1.25	0.13

### Defective oxidative stress response leads to increased susceptibility to fluoroquinolone antibiotics in Δ*arcR* mutant

The tolerance of *S. aureus* to fluoroquinolones is usually associated with mutations of topoisomerase II genes, including *gyrA* and *gyrB*, and high expression of NorA, NorB, NorC, and SdrM belonging to MFS superfamily efflux pump (Lowy, 2003; Ding et al., 2008; Xu et al., 2011). Expression of *norA*, *norB*, *norC*, and *sdrM* was detected in Δ*arcR* mutant, and there was no significant difference compared with the wild-type strain (Supplementary Figures 1A–D). These results suggest that ArcR influenced the resistance of *S. aureus* against fluoroquinolone antibiotics through other mechanisms.

**Figure 2:**
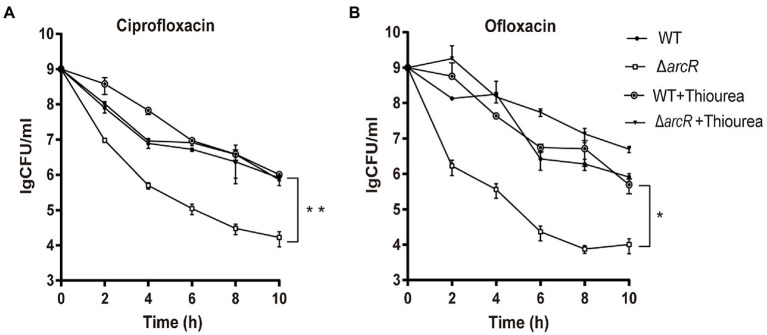
Effects of hydroxyl radical scavenger on bacterial resistance to fluoroquinolone antibiotics. Bacteria were treated with **(A)** 0.32 μg/ml ciprofloxacin or **(B)** 0.5 μg/ml ofloxacin in the presence or absence of 150 mM thiourea in TSB at 37°C with agitation. At the indicated times, the live bacterial counts were determined by serial dilution and plating. Error bars represent standard deviations. The data represent results from three independent experiments. *p < 0.05, **p < 0.01, and ***p < 0.001 by Student’s *t*-test.

It was reported that ROS was involved in the bactericidal process of aminoglycoside, β-lactam, and fluoroquinolone antibiotics (Kohanski et al., 2007). To determine the role of ROS in the increased sensitivity of Δ*arcR* mutant to fluoroquinolones, thiourea that neutralize intracellular ROS was added into the medium when killing curves were detected. Thiourea supplementation increased the survival rate of the Δ*arcR* mutant strain to a level similar to that of the wild type when treated with the same concentration of antibiotics (Figures 2A,B). These results suggest that a defective oxidative stress response leads to increased sensitivity to fluoroquinolone antibiotics in Δ*arcR* mutant.

### ArcR is involved in bacterial tolerance to oxidative stress

**Figure 3:**
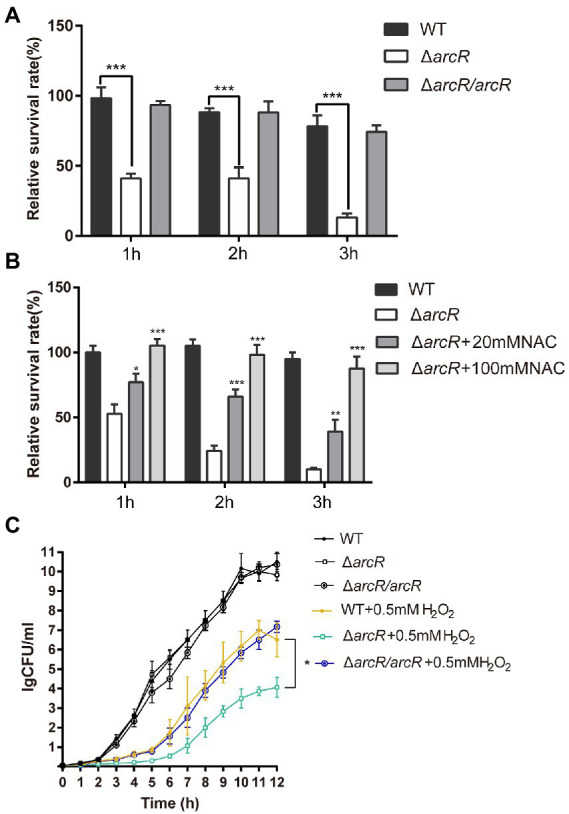
Roles of ArcR in bacterial tolerance to neutrophils and H_2_O_2_. **(A)** Bacteria were incubated with mouse neutrophils at an MOI of 5 in HBSS or **(B)** HBSS containing 80 mM NAC. Live bacterial counts were determined every hour for 3 h. **(C)** Bacteria were treated with 0.4 mM H_2_O_2_ for 12 h in TSB at 37°C. Live bacterial counts were determined by serial dilution and plating. The survival rate was calculated as the number of CFUs of each strain after individual treatment divided by the number of CFUs in the corresponding sample before treatment. The data represent results from three independent experiments. *p < 0.05, **p < 0.01, and ***p < 0.001, by Student’s *t*-test.

The bacterial response to oxidative stress is a manifestation of virulence, which can help bacteria to resist ROS produced by phagocytes and enhance their infectivity. Activated neutrophils produce O_2_•^−^, H_2_O_2_, nitric oxide (NO), and hypochlorite (HOCl), which kill invading pathogenic bacteria through oxidative burst (Forman et al., 2002; van der Veen et al., 2009). To understand the role of ArcR in the bacterial oxidative stress response, bacteria were incubated with neutrophils. The wild type and Δ*arcR* mutant could introduce the same level ROS produced by neutrophils when they were incubated with neutrophils (Supplementary Figure 2). However, the survival rate of Δ*arcR* mutant was significantly lower than that of wild-type strain when they were treated with neutrophils (Figure 3A). The presence of ROS-neutralizing N-acetylcysteine (NAC) increased the survival rate of Δ*arcR* mutant after incubation with neutrophils (Figure 3B). We used H_2_O_2_ to verify the role of ArcR in bacterial tolerance to oxidative stress, and the Δ*arcR* mutant showed higher sensitivity to H_2_O_2_ than wild-type strain (Figure 3C). These results suggest that ArcR contributes to bacterial resistance to oxidative stress.

### ArcR participates in regulation of oxidative stress resistance genes

**Figure 4:**
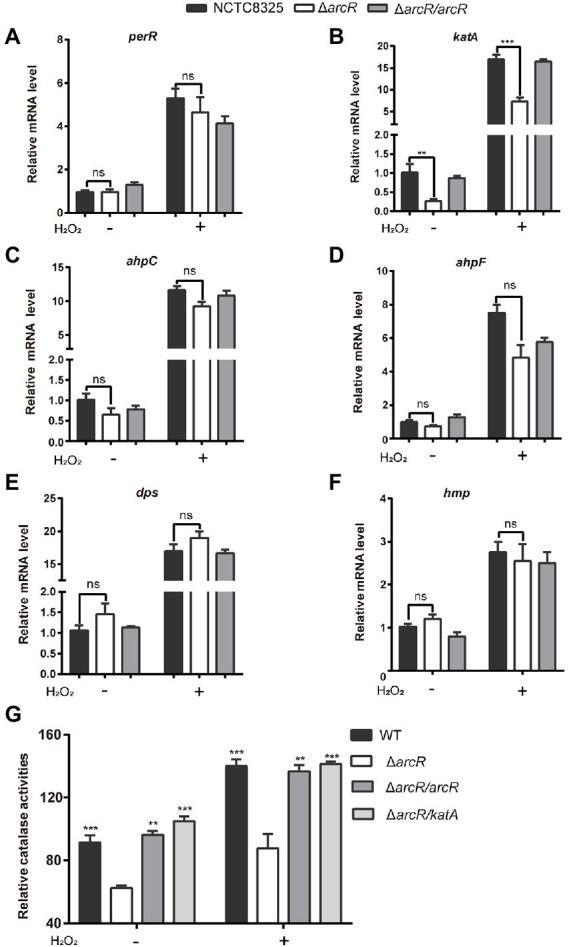
Expression of oxidative stress responsive genes. Bacteria were treated with or without 0.4 mM H_2_O_2_ for 10 min in PBS. The mRNA levels of *perR* **(A)**, *katA* **(B)**, *ahpC* **(C)**, *ahpF* **(D)**, *dpS* **(E)**, and *hmp* **(F)** were determined by real-time PCR. The 16S rRNA gene was used as an internal control. **(G)** Bacteria were lysed by sonication, and catalase activity was determined with a catalase assay kit. The data represent results from three independent experiments. ns, not significant. *p < 0.05, **p < 0.01, and ***p < 0.001, by Student’s *t*-test.

The increased sensitivity to ROS suggested that the expression of genes involved in ROS response might be reduced in Δ*arcR* mutant. PerR is a major global regulator of H_2_O_2_ response, which is a repressor of peroxide reaction (Dubbs and Mongkolsuk, 2012). The expression of PerR was not significantly altered in Δ*arcR* mutant (Figure 4A). We measured the expression of other ROS resistance genes. In the presence or absence of H_2_O_2_, mRNA levels of *katA*, which encodes primary catalase, were approximately fourfold lower in the Δ*arcR* mutant than in the wild-type strain (Figure 4B). Whereas, the mRNA levels of *ahpC*, *ahpF*, *dps*, and *hmp* were similar between the wild-type and Δ*arcR* mutant (Figures 4C–F). Total catalase activity was lower in Δ*arcR* mutant than that in wild-type strains when treated with or without H_2_O_2_, which consistent with the change of *katA* mRNA levels (Figure 4G). Complement with *arcR* or *katA* in Δ*arcR* mutant restored the catalase to wild-type level (Figure 4G).

**Figure 5:**
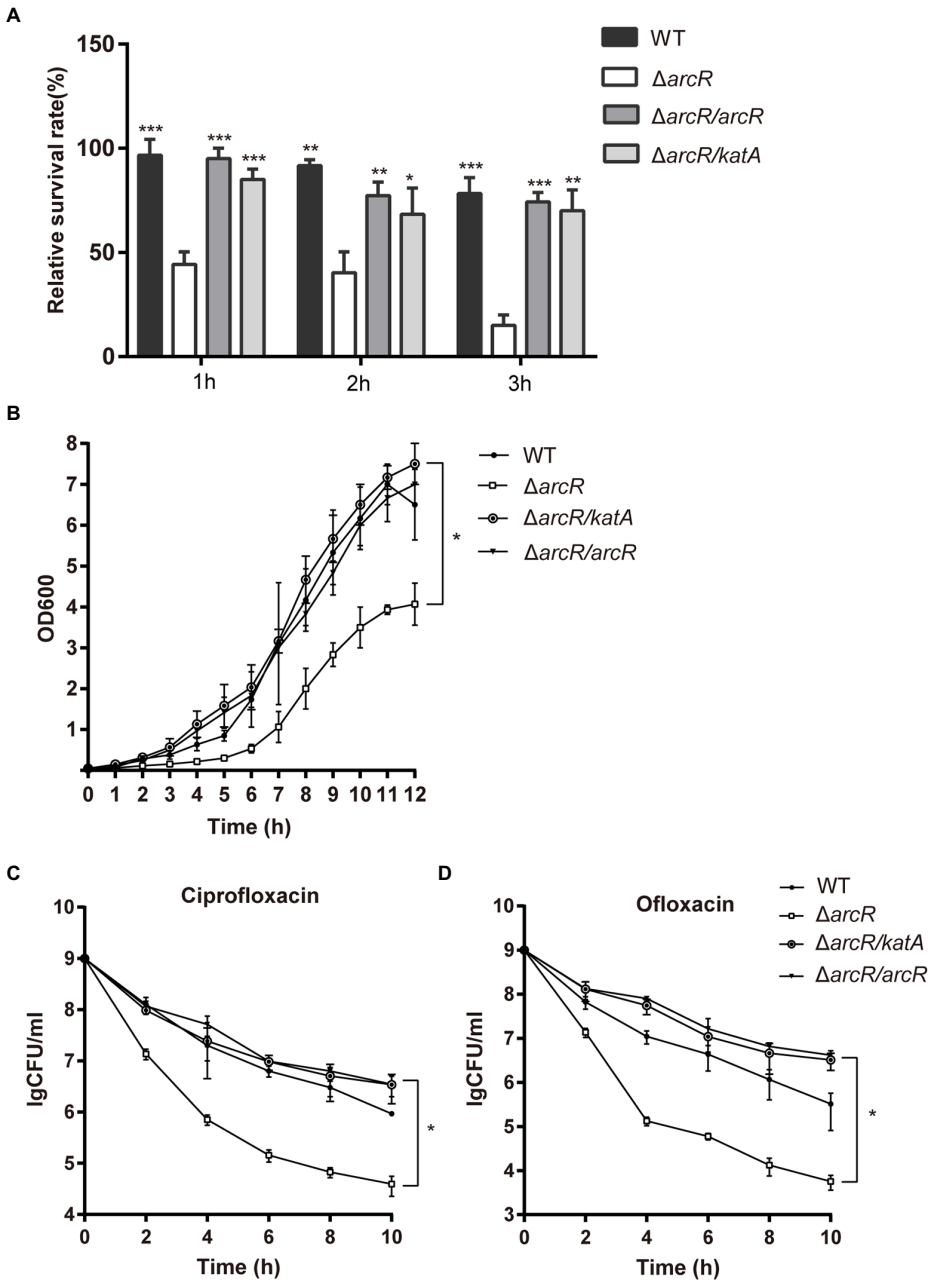
Overexpression of *katA* restored resistance of Δ*arcR* mutant against antibiotics and oxidative stress. **(A)** Bacteria were incubated with mouse neutrophils at MOI 5 in HBSS for 3 h. **(B)** Bacteria were treated with 0.4 mM H_2_O_2_ for 12 h in TSB at 37°C. (C and D) Bacteria were treated with **(C)** 0.32 μg/ml ciprofloxacin or **(D)** 0.5 μg/ml ofloxacin for 10 h in TSB with agitation. Live counts were determined by plating. Δ*arcR*/*katA* represented the *katA* overexpression strain, pCN51-*katA* in Δ*arcR* mutant. The data represent results from three independent experiments. ns, not significant. *p < 0.05, **p < 0.01, and ***p < 0.001, by Student’s *t*-test.

Overexpression of *katA* in Δ*arcR* mutant strain restored bacterial tolerance to neutrophils, H_2_O_2_, ofloxacin, and ciprofloxacin (Figures 5A–D). These results confirmed that downregulation of *katA* expression was responsible for bacterial susceptibility to H_2_O_2_ which increased Fenton reaction and OH• production and finally resulted in decreased tolerance to fluoroquinolone antibiotics.

### ArcR promotes expression of *katA* at the transcriptional level by directly binding to the *katA* promoter region

**Figure 6:**
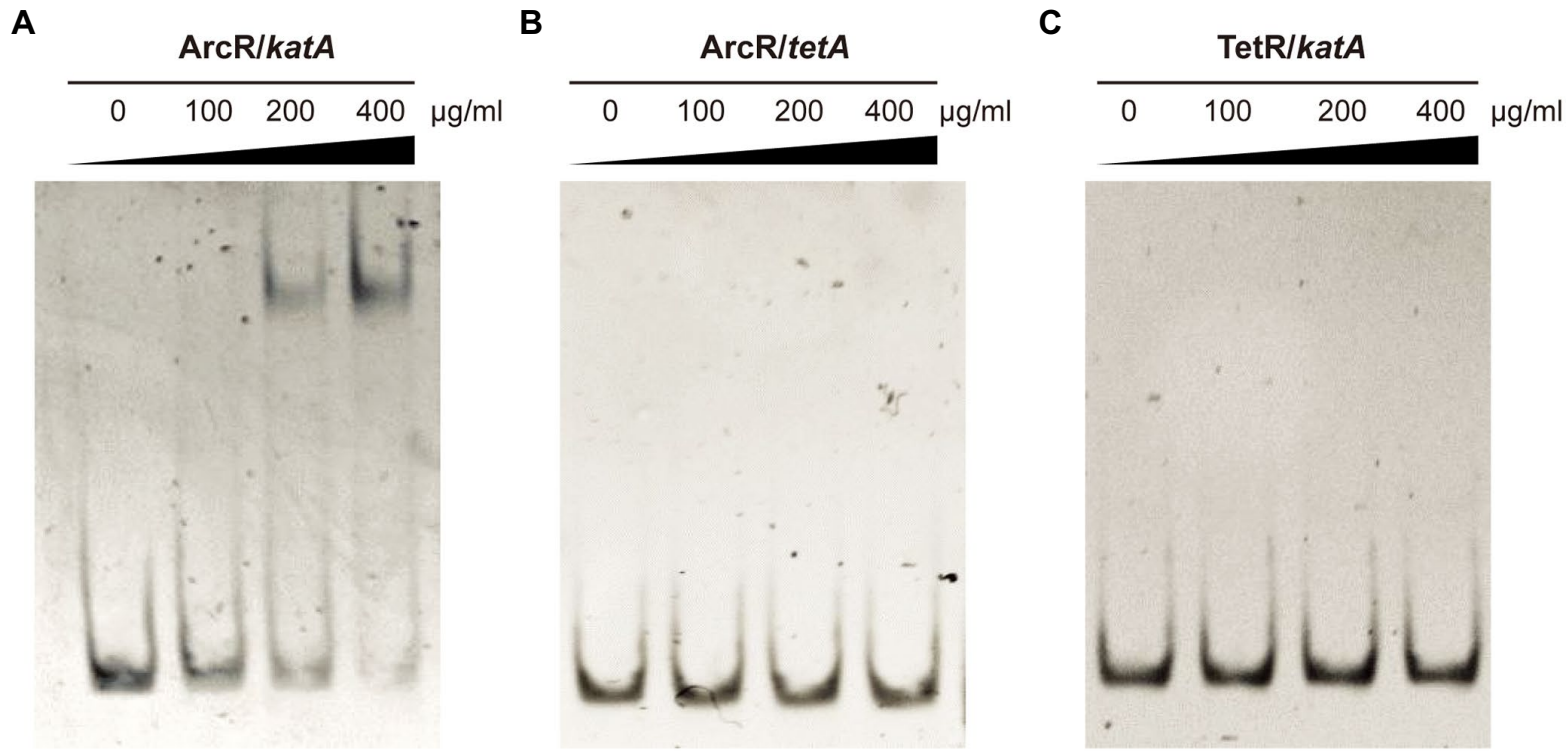
ArcR promotes expression of *katA* by directly binding to its promoter region. Interaction between ArcR and *katA* promoter region was examined by EMSA. ArcR was incubated with *katA* promoter region **(A)**, ArcR was incubated with *tetA* promoter region **(B)**, and TetR was incubated with *katA* promoter region **(C)** at 37°C for 30 min. The mixtures were electrophoresed on agarose gels and the bands were visualized under UV light after ethidium bromide staining. The data represent results from three independent experiments.

The ArcR regulator of *S. aureus* contains two domains that are characteristic of members of the Crp family of regulatory proteins. Structural prediction showed that the N terminus of ArcR had a circular nucleotide binding domain, and the C terminus contained a highly conserved helix-turn-helix DNA-binding domain. To explore how ArcR regulated *katA* transcription, we used pET 28a to express ArcR protein in *E. coli* BL21. Electrophoretic mobility shift assay (EMSA) was performed to detect the binding of ArcR to 244-bp DNA in the promoter region of *katA*. The amount of free DNA gradually decreased as the amount of ArcR protein increased (Figure 6A). The specificity of binding was reflected in the fact that DNA mobility was not affected when an unrelated His-tagged TetR protein was used. It also did not bind to 267-bp DNA of *tetA* promoter region (Figures 6B,C). These results suggested that the binding of ArcR to the *katA* promoter region was specific.

## Discussion

We found that ArcR controlled the resistance of bacteria to fluoroquinolones by modulating catalase KatA. Enzymes involved in the oxidative stress response have been shown to contribute to antibiotic resistance. KatA and superoxide dismutase are involved in bacterial antibiotic resistance. In *Enterococcus faecalis*, the absence of superoxide dismutase gene *sodA* reduced the bacterial tolerance to vancomycin and penicillin (Bizzini et al., 2009). In *Acinetobacter baumannii*, the *sod2343* mutant strains were more susceptible to colistin and tetracycline (Heindorf et al., 2014). In *Pseudomonas aeruginosa*, *katA* plays an important role in bacterial tolerance to aminoglycoside and β-lactam antibiotics (Xia et al., 2019). KatA is the major catalase in *S. aureus*. Here, we demonstrated that KatA was involved in bacterial tolerance to oxidative stress and subsequent tolerance to fluoroquinolone antibiotics. Additionally, KatA affected the survival of *S. aureus* at low temperature (Masmoudi et al., 2010; Suo et al., 2022).

PerR functions as a major repressed regulator of the response to H_2_O_2_ in *S. aureus*, which replaces OxyR in many Gram-positive bacteria (Mongkolsuk and Helmann, 2002; Dubbs and Mongkolsuk, 2012). In *S. aureus*, Fur is a regulator interacting with PerR, which participates in the oxidative stress response by positively regulating catalase and iron homeostasis (Horsburgh et al., 2001). Sigma B controls the general stress response, and there are sigma B recognition sites in the *katA* promoter region, which in some cases regulate *katA* expression (Horsburgh et al., 2002). ppGpp regulates *katA* expression in a PerR-independent manner (Horvatek et al., 2020). The *srrAB* two-component regulatory system can inhibit expression of *katA*, and the *srrA* promoter region is predicted by bioinformatics to contain an ArcR binding site (Makhlin et al., 2007; Oogai et al., 2016). To determine whether ArcR also regulated expression of *katA* by repressing *srrAB*, we detected expression of *srrAB* in Δ*arcR* mutant by quantitative RT-PCR and found that it was not significantly different from that in the wild-type strain. This indicates that ArcR directly regulates expression of *katA*, and this process is independent of *srrAB*.

Glucose in combination with aminoglycosides could be used to treat the biofilms of *E. coli* and *S. aureus*. Glucose catabolism generated NADH and then NADH was oxidized in the electron transport chain, which, in turn, contributed to PMF. The elevated PMF facilitated the uptake of aminoglycoside antibiotics (Allison et al., 2011; Peng et al., 2015). Cyclic lipopeptide resistance can also be affected by glucose, which induces bacterial resistance to polymyxin B by enhancing glycolytic flux to maintain intracellular ATP levels in PB-treated bacteria. Similar results were observed in *S. aureus*, where glucose enhanced daptomycin resistance (Yu et al., 2019). In *S. aureus*, glucose could inhibit the expression of *arcR*. Meanwhile, *arcR* affect the response of *S. aureus* to ROS and then affect the sensitivity to fluoroquinolone antibiotics. Therefore, glucose most likely influences the tolerance of *S. aureus* to fluoroquinolone antibiotics through ArcR. This study may add to our understanding on the role of carbon metabolism in bacterial susceptibility to antibiotics.

## Data availability statement

The raw data supporting the conclusions of this article will be made available by the authors, without undue reservation.

## Author contributions

TF, ZF, and YL designed the experiments, performed the experiments, and wrote the manuscript. JY designed the experiments and revised the manuscript. All authors performed the experiments, analyzed the results, or revised the manuscript. All authors contributed to the article and approved the submitted version.

## Funding

This work was supported by grants from the National Natural Science Foundation for Key Programs of China Grants (82130065), National Natural Science Foundation of China (32170201 and 82002191), Beijing Natural Science Foundation (7222014), FENG foundation (FFBR 202103), the Research Foundation of Capital Institute of Pediatrics (CXYJ-2021-04), Public service development and reform pilot project of the Beijing Medical Research Institute (BMR2019-11), and Postdoctoral Research Fund of Chaoyang District, Beijing, China in 2021.

## Conflict of interest

The authors declare that the research was conducted in the absence of any commercial or financial relationships that could be construed as a potential conflict of interest.

## Publisher’s note

All claims expressed in this article are solely those of the authors and do not necessarily represent those of their affiliated organizations, or those of the publisher, the editors and the reviewers. Any product that may be evaluated in this article, or claim that may be made by its manufacturer, is not guaranteed or endorsed by the publisher.
