# Supplementary material for: ArcR contributes to tolerance to fluoroquinolone antibiotics by regulating katA in Staphylococcus aureus
Source: Front Microbiol. 2023 Feb 24;14:1106340. doi: 10.3389/fmicb.2023.1106340 (PMC9998937; doi:10.3389/fmicb.2023.1106340)
Supplement: Supplementary file 1 [file Data_Sheet_1.doc]

**
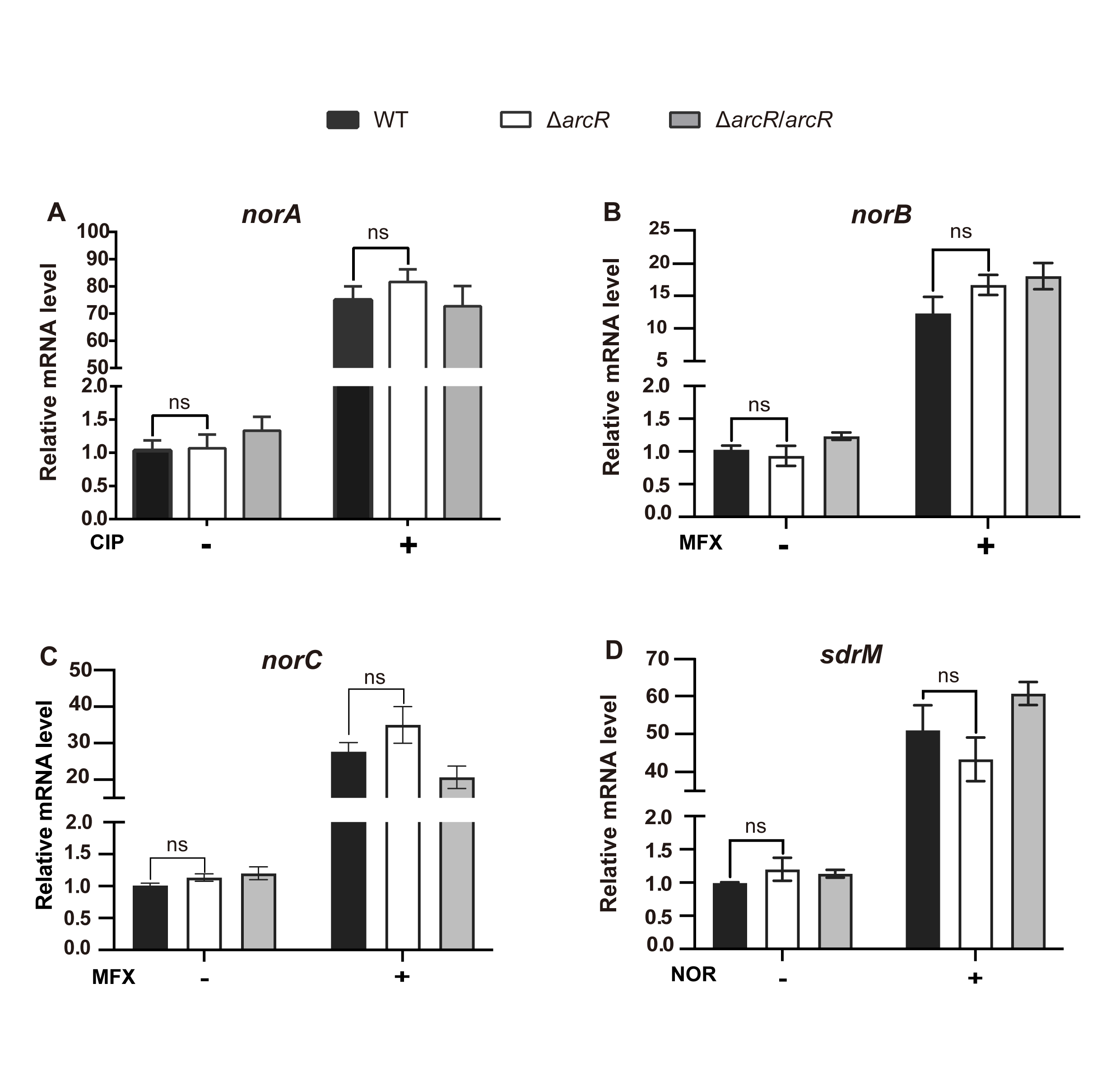
**

**Supplementary Figure 1 Expression of fluoroquinolone-resistance-related genes.**

Bacteria were treated with or without 0.3 μg/ml ciprofloxacin, 0.5 μg/ml norfloxacin or 0.2 μg/ml moxifloxacin for 30 min. The relative mRNA levels of (A) *norA*, (B)*norB*, (C) *norC* and (D) *sdrM* were detected by quantitative RT-PCR. The 16S ribosomal RNA gene was used as an internal control. CIP, ciprofloxacin; MFX, moxifloxacin; NOR, norfloxacin; ns, not significant, by Student’s *t* test.

**
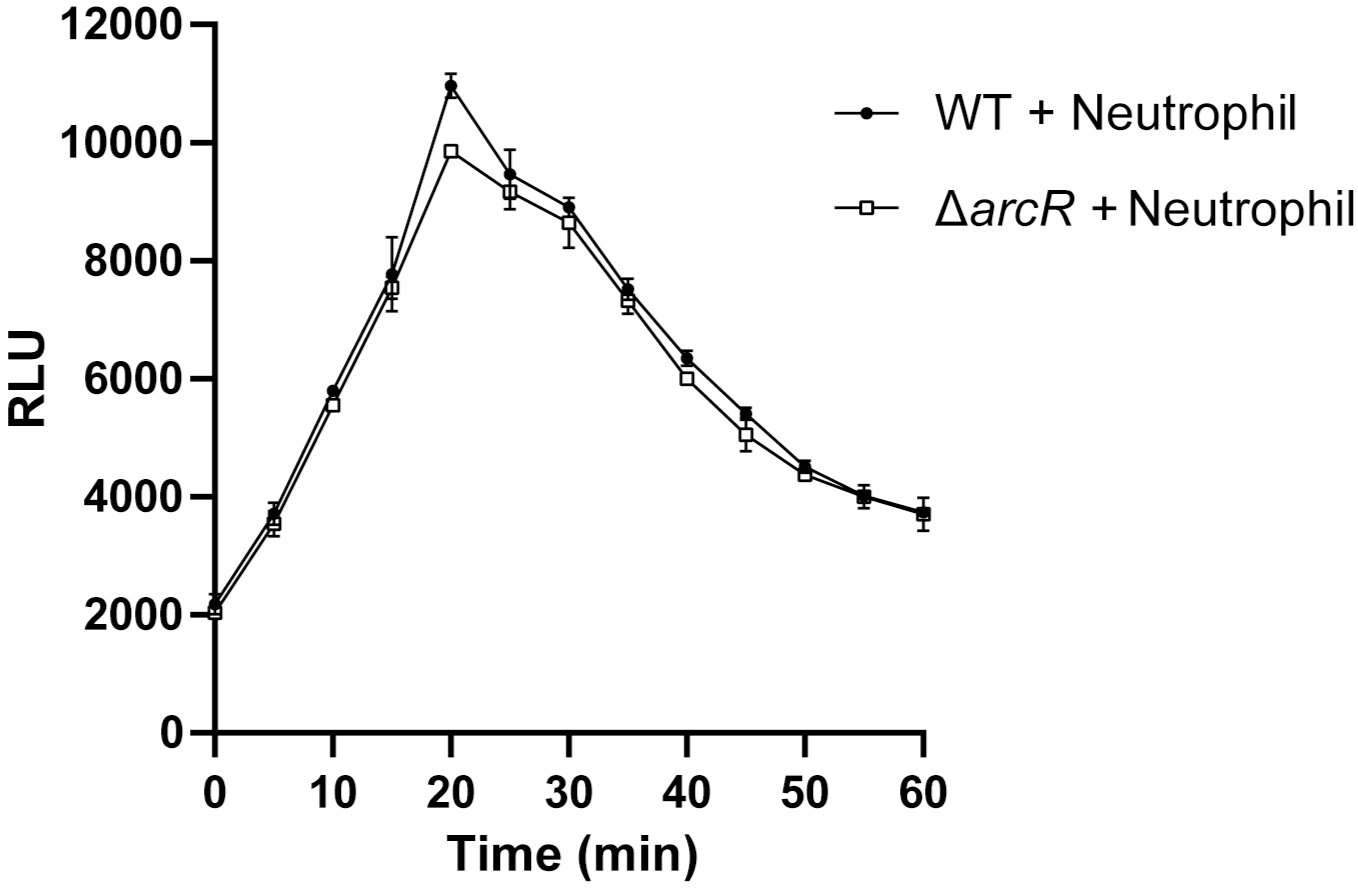
**

**Supplementary Figure 2 ROS production by neutrophils.**

WT or Δ*arcR* mutant were grown to OD_600_ 1.0. Bacteria were incubated with neutrophils at MOI of 5 at 37°C. The relative light units (RLU) were detected every 5 min for 1 h. Error bars represent standard deviations.
